# Determinants of bone damage: An ex-vivo study on porcine vertebrae

**DOI:** 10.1371/journal.pone.0202210

**Published:** 2018-08-16

**Authors:** Mohammad J. Mirzaali, Flavia Libonati, Davide Ferrario, Luca Rinaudo, Carmelo Messina, Fabio M. Ulivieri, Bruno M. Cesana, Matteo Strano, Laura Vergani

**Affiliations:** 1 Department of Mechanical Engineering, Politecnico di Milano, Milan, Italy; 2 TECHNOLOGIC S.r.l. Hologic Italia, Torino, Italy; 3 Istituto Ortopedico Galeazzi IRCCS, Radiodiagnostic Unit, Milan, Italy; 4 Fondazione IRCCS Cà Granda Ospedale Maggiore Policlinico, Nuclear Medicine-Bone Metabolic Unit, Milan, Italy; 5 Department of Clinical Sciences and Community Health, Unit of Medical Statistics, Biometry and Bioinformatics "Giulio A. Maccacaro", Faculty of Medicine and Surgery, University of Milan, Milan, Italy; Rensselaer Polytechnic Institute, UNITED STATES

## Abstract

Bone’s resistance to fracture depends on several factors, such as bone mass, microarchitecture, and tissue material properties. The clinical assessment of bone strength is generally performed by Dual-X Ray Photon Absorptiometry (DXA), measuring bone mineral density (BMD) and trabecular bone score (TBS). Although it is considered the major predictor of bone strength, BMD only accounts for about 70% of fragility fractures, while the remaining 30% could be described by bone “quality” impairment parameters, mainly related to tissue microarchitecture. The assessment of bone microarchitecture generally requires more invasive techniques, which are not applicable in routine clinical practice, or X-Ray based imaging techniques, requiring a longer post-processing. Another important aspect is the presence of local damage in the bony tissue that may also affect the prediction of bone strength and fracture risk. To provide a more comprehensive analysis of bone quality and quantity, and to assess the effect of damage, here we adopt a framework that includes clinical, morphological, and mechanical analyses, carried out by means of DXA, μCT and mechanical compressive testing, respectively. This study has been carried out on trabecular bones, taken from porcine trabecular vertebrae, for the similarity with human lumbar spine. This study confirms that no single method can provide a complete characterization of bone tissue, and the combination of complementary characterization techniques is required for an accurate and exhaustive description of bone status. BMD and TBS have shown to be complementary parameters to assess bone strength, the former assessing the bone quantity and resistance to damage, and the latter the bone quality and the presence of damage accumulation without being able to predict the risk of fracture.

## 1. Introduction

Osteoporosis is a metabolic bone disease characterized by low bone mass and micro-architectural deterioration of bone tissue, leading to enhanced bone fragility and a consequent increase in fracture risk [1]. Osteoporosis also presents a compromised bone strength, as this mechanical property reflects the integration of bone quantity (measured as bone mineral density, BMD) and bone quality (an index related to bone microarchitecture, bone geometry and bone turnover) [1].

BMD is the major predictor of bone resistance to load and fracture, and Dual-X Ray Photon Absorptiometry (DXA) is the gold standard method to measure bone “quantity” [2–4]. Despite its primary role in assessing bone strength, BMD only accounts for about 70% of fragility fractures, while the remaining 30% could be explained by the impairment of bone “quality” parameters that are the geometric and material factors contributing to fracture resistance independently of bone mineral density [3,5,6]. BMD is, indeed, limited by its two-dimensional nature, being areal measurement, and it can not capture the three-dimensional microarchitecture, considered as a key determinant of bone strength [5,7]. Moreover, it is affected by the size and position of the subject [8] and cannot distinguish between the cortical and trabecular compartments. Novel and useful information on the bone microarchitecture can be obtained from the trabecular bone score (TBS) [9–14], a relatively new morphological parameter that can be quickly calculated from DXA (i.e., from 2D-lumbar spine DXA images). In fact, TBS represents a textural measurement, which can provide skeletal qualitative information not obtained by BMD. TBS is based on the experimental variograms of projected gray-level DXA images as reported in [15].

To directly assess the tissue microarchitecture *in vivo* and get high-resolution structural bone parameters, the only available method is the High-resolution peripheral quantitative computed tomography (HR-pQCT) [16–18]. However, it is expensive, not generally available in hospitals, and only applicable to limited sites. The biopsy could be an alternative method for direct assessment of bone microarchitecture [19,20]. It is an invasive procedure though, and does not necessarily reflect the microstructure at sites where the fragility fractures occur, like spine and femur.

The most important parameters, which could be used to improve the clinical assessment of bone fragility, are morphological parameters, such as relative bone density (ρ_s_), also measured as Bone Volume to Total Volume (BV/TV), trabecular thickness (Tb.Th), trabecular spacing (Tb.Sp), trabecular number (Tb.N.) [21–23], and the degree of anisotropy (DA) [24–27]. These parameters can be calculated by means of high-resolution imaging techniques such as micro-computed tomography (μCT). μCT-scanning allows one to get the three-dimensional image of the trabecular bone microstructure, whereas ad hoc post-processing techniques allow one to estimate the orientation and distribution of the trabeculae [28].

The relationship between TBS texture parameters and 3D-microarchitecture parameters has been exhaustively studied in the literature [14]. Several ex vivo studies have reported significant correlations between TBS and various bone microstructural parameters assessed by μCT [14,29–31]. In particular, it has been demonstrated that high TBS values correlate with better skeletal texture, as a reflection of higher connectivity density (the manner of how bone trabeculae are overconnected in 3D volume) [32], whereas low TBS values correlate with weaker skeletal texture, as a reflection of degraded micro-architecture [13], and with an increase in prevalent and incident fractures [10]. Besides, recent studies have proved that TBS can discriminate and predict fragility fractures, together with BMD, both in primary and in secondary osteoporosis [33,34].

The effective validity of all the measured morphological and clinical parameters depends on their accuracy as predictors of the mechanical properties of bone, such as compressive strength and elastic stiffness. Several studies have been carried out to find the correlations between DXA, morphological parameters, and the mechanical characteristics. In particular, it has been shown that there is a correlation between BMD and mechanical parameters such as elastic stiffness, strength, and maximum energy absorption [7]. Elastic stiffness has shown to be correlated to TBS [35], and a significant correlation between morphometric and mechanical parameters has also been stated [36,37].

Although most of the studies have been focused on the investigation of the relationship between clinical-morphological parameters and the mechanical ones [38–40], a systematic investigation on the effect of mechanical damage is needed. Vertebral fractures in non-traumatic events are the consequences of damage accumulation and permanent deformation in the vertebral body [41,42]. The accumulation of damage forms microcracks [43–45], which are not detectable by clinical radiographs [46,47]. However, they result in degradation of stiffness and strength and permanent residual strains [41,48–50]. Accumulation of damage in trabecular bone is dependent on site, density, and species [38,48,51–54], and loading mode [55–57]. To validate the use of morphological and clinical parameters as predictors of the effective mechanical properties of bone and fracture risk, also taking into account the potential presence of local damage, a methodical multidisciplinary study, involving the expertise of engineers and medical doctors, is needed.

Considering the current state of the art, the proposed study aims to get an insight into the degradation of bone from a mechanistic point of view, with the goals of determining the sensitivity of the clinical and morphological parameters to the damage accumulation, and their ability in measuring the damage and the risk of fracture. To reach these goals, providing a more comprehensive analysis of bone quality, quantity and damage, we propose a framework that includes clinical, morphological, and mechanical analyses, carried out by means of DXA, μCT and mechanical compressive testing, respectively. DXA allowed us to measure the bone quality in terms of mineral content, mechanical compressive testing allowed us to have a quantitative indication of strength and damage, whereas μCT provided important information about the microarchitecture and its mechanically-induced degradation. This study has been carried out on porcine trabecular bones, as the alignment of the trabeculae, their structural homogeneity and the distribution of the mechanical strength are similar to those of human lumbar spine [37,58].

## 2. Material and methods

**Fig 1:**
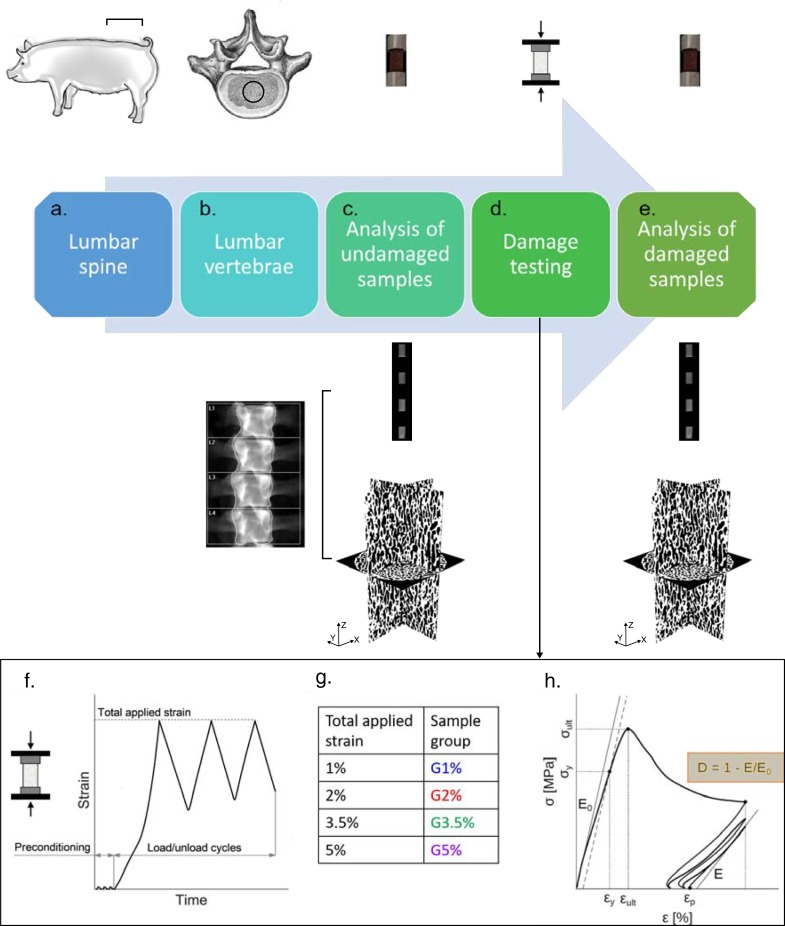
Schematic of the experimental framework adopted. a) Six lumbar vertebrae were taken from pigs and kept frozen. b) DXA was performed on each lumbar body. c) Cylindrical porcine specimens were cut from the core of each lumbar vertebra. DXA scanning was performed for four samples, simultaneously, to get clinical parameters. Then, μCT scanning was performed on each sample to get 3D morphological parameters. d) Porcine specimens were divided into four groups, corresponding to different damage levels, and mechanical compressive tests were performed in each cluster. e) DXA and μCT-scanning were performed on damaged specimens, and corresponding clinical and morphological post-damage parameters were calculated. f) Compressive testing setup and load application: initial preconditioning, followed by three load/unload cycles until a prefixed strain level. g) Nomenclature of the four groups and corresponding total applied strain. h) Schematic of the resulting stress-strain curve and the mechanical properties determined. Damage was calculated as stiffness degradation, as indicated in the yellow box.

We adopted a combination of different characterization techniques. Fig 1 summarizes the framework followed, composed of the following steps:

- Collection of lumbar vertebrae from 6 lumbar spines;
- DXA analysis of lumbar spines;
- Sample preparation;
- Analysis of undamaged samples obtained from lumbar vertebrae through:
      c1) DXA;
      c2) μCT;
- Damage testing of specimens;
- Analysis of damaged specimens through:
      e1) DXA;
      e2) μCT.

Each step is explained in detail in the following.

### 2.1. DXA Scanning of lumbar spines

Porcine lumbar spines were evaluated with a Hologic Discovery A system (Hologic Inc, Marlborough, Massachusetts, USA) installed at the Bone Metabolic Unit of the Nuclear Medicine of the Fondazione IRCCS Ca' Granda-Ospedale Maggiore Policlinico, Milan, Italy. To assess BMD, we used APEX software installed on the same machine, whereas TBS has been calculated automatically by software provided by Medimaps Group, Wilmington, US, and installed on the same machine. DXA image resolution was set to 0.5 mm, and each spine was placed in accordance to the correct anatomical planes. For each spine, after manually removing the residual ribs left from the butcher, we performed a lumbar scan, manually selecting the region of interest, to calculate TBS for each vertebra. We performed the DXA scanning of each full spine, after removal from the animal, before the sample preparation, to make sure that the clinical parameters of the porcine spines were similar to those of adult human vertebrae, according to the International Society of Clinical Densitometry (https://www.iscd.org/ (April 2016)) and to the National Health and Nutrition Examination Survey (NHANES) [59]. Being the segmentation method in DXA scanning a manual procedure, we tested each vertebra three times to calculate the segmentation effect on our results.

### 2.2. Sample preparation

Porcine trabecular specimens were cut from six different vertebral columns. The spines involved six lumbar vertebrae from L1 to L6, except for three, where the L6 was missing. At least one specimen was obtained from each lumbar vertebra, leading to a total of 40 samples tested. However, for the following statistical analyses, the duplicates were removed, because two samples extracted from the same vertebra cannot be considered independent from each other. This led to a total of 33 samples. The animals were one year old and porcine lumbar spine vertebrae were provided from a local butcher (“Salumificio Venegoni S.p.A." a store located in Boffalora Sopra Ticino (MI). The vertebral columns were intended for retail trade by the butcher) and then stored at -18°C till the preparation of the samples and the experimental tests. Samples were drilled using a core drilling device (inner diameter of 16 mm and 40 mm of length) along the anatomical direction of the vertebral column. Subsequently, the samples were transferred to a lathing machine to reduce them to cylinders with the diameter of 13.8 mm and height of 30 mm. During the drilling and turning, the specimens were kept wet by adding water. Then, to reduce the edge effects [60] and eliminate the local damage effect, the ends of the bone samples were glued (3P Scotch-WeldTM EPXTM Adhesive DP490) in custom-made aluminum end caps. The end caps had an inside diameter of 14 mm, outside diameter of 20 mm and height of 15 mm and they covered 3 mm of the specimens on the parallel sides [61]. To obtain perfectly parallel surfaces, both ends of bone specimens were smoothed using a circular blade saw. The bone samples and the aluminum tubes were defatted using acetone before gluing. A custom-made alignment tool was used to keep the bone and the end caps aligned within the direction of axial loading. Specimens were kept frozen at -18°C, and then rehydrated in saline solution (NaCl 0.9%) at 4°C for 12 h before mechanical testing.

### 2.3. Sample imaging

We performed the same scans and analyses on the cylinder samples before and after mechanical testing. In each test, four cylindrical specimens were placed in the machine to perform the DXA scanning, keeping the orientation of the specimens similar to the complete vertebrae. Scanning lasted for about two minutes, and samples were kept frozen before and after scanning, to prevent any deterioration of the microstructure of the bones.

Micro-computed tomography (μCT) images of trabecular specimens were collected using an X-ray Metrology CT system (X25, North Star Imaging Inc., Buckinghamshire, UK) with the spatial resolution of about 25.6 μm. Parameters of the scanning were fixed at 60 kV and 150 μA. Simultaneously, three specimens were placed in the CT equipment and total imaging time was 110 minutes. Specimens were submerged in saline solution during the scanning.

Image reconstruction was performed with the x-view CT software. Image analysis has been performed in ImageJ [62] software and BoneJ plugin [63]. The noise was removed from the images, by using a Gaussian blur filter (standard deviation of the Gaussian distribution 1.5). Thereafter, the images were converted to gray-level 8-bit images. The Otsu local thresholding method [64] was used for the segmentation of the images, resulting in binary images with the voxel value of 1, for bone, and 0, for the empty spaces. Porosity (*ρ*_*p*_) was defined as the total number of cavities to the total number of voxels in ROI (region of interest) in the binary image. The relative density was defined as *ρ*_*s*_ = 1 – *ρ*_*p*_. Trabecular thickness (Tb.Th) and trabecular spacing (Tb. Sp) were calculated based on the conventional definition of the greatest sphere diameter that fits within the structure [65]. Bone surface (BS) was defined as the inside surfaces of the bone materials and was calculated by the isosurface creation of the binary image with the resampling equal to 1 [66].

The main direction of the microstructures of the bones was measured by Mean Intercept Length (MIL) [27]. It has been shown that an ellipsoid can approximate MIL in three-dimensions [25], and lead to the definition of a positive definite second-order fabric tensor that characterizes the degree of anisotropy of the bone microstructures. Moreover, based on the general theory, developed by [67], the fabric tensor, which is the inverse of MIL tensor, has been applied to measure the local structural anisotropy. Based on the distinct eigenvalues, the fabric can be isotropic, transversely isotropic or orthotropic [68]. The degree of anisotropy (*DA*) can be defined as the complement to the unity of the ratio of the smallest over the largest fabric eigenvalue $DA=1-\frac{\min\left(m_{i}\right)}{\max\left(m_{i}\right)}.$

### 2.4. Mechanical damage tests

Monotonic compression tests were carried out in an MTS machine (Alliance, RF/150) with a load cell of 150 kN (class 1 ISO 7500–1). The specimens were loaded in displacement control along the central cylindrical axis. The axial strain was measured using an extensometer (MTS 632.26F-20 with 8 mm gauge length) attached to the sample. The quasi-static test was performed at a strain rate of 0.0002 s^-1^ (constant stroke rate of 0.05 mm/s). A schematic of the testing protocol adopted is provided in Fig 1F–1H. The loading protocol contained three preconditioning compression cycles up to 0.1% axial strain, followed by monotonic loading until certain strain levels. After that, the specimens were unloaded and loaded three times until the same strain level, to obtain damage and residual plastic strain (Fig 1F). The specimens were divided into four groups, each set loaded until reaching a different strain value. In particular:

- Group 1, called *G1%*, which includes the specimens loaded until 1% of strain, close to the yield strain;
- Group 2, called *G2%*, which includes the specimens loaded until 2% of strain, close to the ultimate strain;
- Group 3, called *G3*.*5%*, which includes the specimens loaded until 3.5% of strain;
- Group 4, called *G5%*, which includes the specimens loaded until 5% of strain.

All the tests were conducted at room temperature. Data were acquired at a sampling rate of 20 Hz. The recording included time (*s*), stroke (*S*), force (*F*), and axial strain (*ε*). Normal stress (*σ*σ) was defined as the ratio of axial force (*F*) to primary area (*A*_0_), obtained from μCT scans for each sample. The initial elastic modulus (*E*_0_) was calculated using a moving regression with a box width of 0.2% strain to identify the stiffest section of the loading part [55,57]. The yield stress (*σ*_*y*_) and yield strain (*ε*_*y*_) were obtained based on a 0.2% offset criterion. The ultimate stress (*σ*_*ult*_) was attained as the maximum primary stress before densification and its corresponding strain as ultimate strain (*ε*_*ult*_). Unloading stiffness (*E*) was calculated from the steepest part of the last loading cycle. Damage (*D*) was defined as:

$$D=1-\frac{E}{E_{0}}$$ (1)

### 2.5. Statistical analysis

An unbalanced Latin Square design was used for mechanical testing. A statistical analysis was carried out in MATLAB^®^ (R2015a) and SAS 9.2, and a p-value < 0.05 was assumed as the significant level. Morphological, clinical and mechanical parameters were analyzed with simple linear regression models to find possible relationships.

ANCOVA was used to test the effect of group, location (fixed factors), and animal (random factor) on the difference of the considered variables between “before” and “after damage”, with the “before damage”-value as the covariate. Multiple pairwise comparisons between the group levels have been carried out by means of the Tukey’s HSD test with the p-value adjusted for multiplicity. In addition, a test of the null hypothesis that the difference between “before” and “after” damage is equal to zero has been carried out on the estimated least squares means of each group with a significance level of 0.0125, according to the Bonferroni’s correction.

## 3. Results

**Fig 2:**
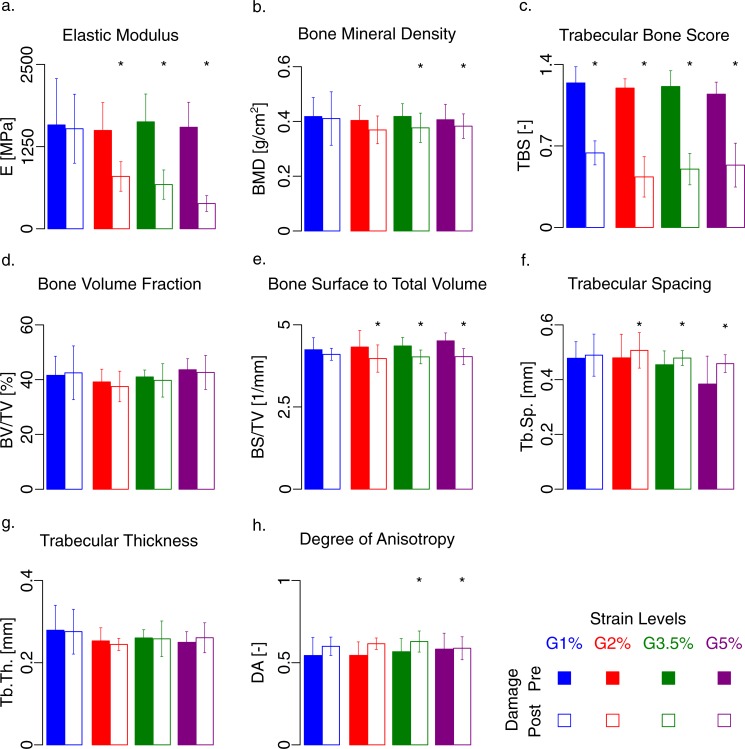
Variation of mechanical (E), clinical (BMD and TBS) and morphological parameters (BV/TV, BS/TV, Tb.Sp, Tb.Th, DA), -before and after damage—for each mechanical damage level (G1%, G2%, G3.5%, and G5%). The filled bars represent the results before damage and the unfilled ones after damage; the labels on the abscissa indicate the damage level. a) Elastic modulus significantly changes within the different group. b) BMD is not affected by the presence of damage. c) TBS significantly changes due to post-yield damage, although no significant difference has been observed between different post-yield damage levels. d) BV/TV does not change through damage loading. The variation of BS/TV (e) and Trabecular spacing (f) is nonzero for the three mechanical groups, G2%, G3.5%, and G5%. g) Trabecular thickness remained the same after applying the damage. h) The degree of anisotropy significantly changes for groups 3.5% and 5%. Asterisk sign shows the significant level (p < 0.0125) in the case of comparison, based on the ANCOVA tests.

Fig 2 shows how the clinical (BMD and TBS), morphological (BV/TV, BS/TV, Tb.Sp, Tb.Th, DA), and mechanical (E) parameters are affected by four different mechanical damage levels (G1%, G2%, G3.5%, and G5%). Table 1 summarizes the adjusted linear correlation between mechanical, morphological and clinical parameters.

**Table 1:** Adjusted linear correlations (R^2^) between mechanical properties, morphological and clinical parameters. In case of significant linear correlation (p < 0.05), the corresponding cells are gray-highlighted. Results are obtained from micro-CT scan before the damage tests.

	BMD	TBS	E_0_	ε_y_	σ_y_	ε_ult_	σ_ult_	BV/TV	BS	BS/BV	BS/TV	TbTh	TbSp	SMI	DA
BMD	1.00	0.03	0.20	-0.03	0.23	-0.03	0.34	0.21	0.02	0.26	-0.03	0.23	-0.03	0.17	-0.03
TBS		1.00	-0.03	0.13	0.10	-0.04	-0.03	0.22	0.07	0.09	0.06	0.03	0.01	0.12	-0.02
E_0_			1.00	0.18	0.42	0.16	0.67	-0.02	0.01	-0.01	-0.03	-0.03	-0.02	0.06	0.09
ε_y_				1.00	0.08	0.01	-0.01	-0.01	0.15	-0.03	-0.01	-0.03	-0.03	-0.02	-0.04
σ_y_					1.00	0.08	0.76	0.07	0.26	0.04	-0.02	-0.02	-0.04	0.17	0.07
ε_ult_						1.00	-0.04	-0.02	-0.04	-0.02	-0.04	-0.03	-0.04	-0.04	-0.05
σ_ult_							1.00	0.09	0.35	0.04	-0.01	-0.01	-0.04	0.18	0.18
BV/TV								1.00	0.10	0.51	0.30	0.21	0.42	0.48	0.07
BS									1.00	-0.02	0.43	0.05	0.10	0.27	0.07
BS/BV										1.00	-0.01	0.56	0.02	0.23	0.10
BS/TV											1.00	0.03	0.43	0.13	-0.03
TbTh												1.00	-0.03	-0.03	0.21
TbSp													1.00	0.14	-0.01
SMI														1.00	0.01
DA															1.00

### 3.1. DXA scanning

As the DXA segmentation was performed manually, we checked the reproducibility of our results by repeating the scans three times. The coefficient of variation is smaller for entire vertebra than for small specimens. Indeed, results show that for the entire vertebra there is a coefficient of variation of 1.64% and 1.31% respectively for BMD and TBS, by considering the average of the results obtained by three measurements. The coefficient of variation measured from the three repetitions of the measurements for the porcine trabecular specimens for BMD and TBS are 10.04% and 10.73%, respectively.

For the entire porcine vertebra, the mean ± standard deviation of BMD and TBS are 1.16 ± 0.12 g/cm^2^ and 1.58 ± 0.08, respectively. The clinical parameters of the individual specimens were significantly different from those of the entire vertebra. Indeed, by reducing the size of the vertebra and producing the cylindrical samples, the mean ± standard deviation will change to 0.41 ± 0.05 g/cm^2^ for BMD and 1.19 ± 0.11 for TBS, as the cortical part of the vertebra is removed for the trabecular specimens.

Clinical parameters (BMD, TBS), were similar between animals and vertebral locations (p > 0.05). Therefore, we were able to pool the results (S1 and S2 Table). We found that BV/TV is linearly related to BMD (R^2^ = 0.21) and to TBS (R^2^ = 0.22). By comparing the clinical and the morphological parameters, we found a linear relationship between BMD and the other morphological parameters, such as BS/BV (R^2^ = 0.26), and Tb. Th (R^2^ = 0.23) (p < 0.01) (Table 1).

By observing the values of BMD and TBS, we can notice how the clinical parameters significantly changed after mechanical loading (p < 0.05). The effect of different damage levels on clinical parameters is depicted in Fig 2B and 2C. In particular, the variation of BMD (Fig 2B) differs from zero for G3.5% and for G5% (p < 0.01). The variation of TBS (Fig 2C) is statistically significant for the four mechanical groups (p < 0.01). However, we could not define a damage parameter able to quantitatively describe the effect of different damage levels on TBS values.

### 3.2. μCT-analyses

The tests showed that all morphological parameters do not depend on the location of the spine and the animal (p > 0.05). Morphological parameters in this study are in the same range of reported values in the literature [69]. Morphometric properties are reported in S3 Table.

We found a linear relationship between BV/TV and different morphological parameters such as BS/BV (R^2^ = 0.51), BS/TV (R^2^ = 0.30), trabecular thickness (Tb.Th) (R^2^ = 0.21) and trabecular separation (Tb.Sp) (R^2^ = 0.42) (p < 0.005).

The correlation between the bone surface (BS) and relative bone volume (BV/TV) for human trabecular bone has already been reported before [70]. From our results, we also found a direct correlation between BS/TV and Tb.Sp (R^2^ = 0.10, p < 0.05). Similar linear correlation was found between BS/TV and BV/TV (R^2^ = 0.10, p < 0.05), as shown in Table 1.

Fig 2D highlights the variation of BV/TV with respect to the undamaged specimens for each damage level. Statistical analysis confirmed no significant reduction of BV/TV neither for each level of damage nor between the damage levels (Fig 2D). BS/TV (Fig 2E) shows a significant reduction for the groups after yield. Tb.Sp significantly varies for G2%, G3.5% and G5% (p < 0.005) (Fig 2F), whereas the variation of DA is significant for G3.5% and for G5% (p < 0.01) (Fig 2H). No significant changes were observed for the other morphological parameters such as Tb.Th (Fig 2G).

### 3.3. Mechanical damage tests

The mechanical properties in the four groups are similar (p > 0.5). Therefore, we pooled these data to calculate the mean value of the mechanical properties (see S4 Table).

Mechanical properties determined from the porcine samples are in the same range of the values reported in the literature [71,72].

**Fig 3:**
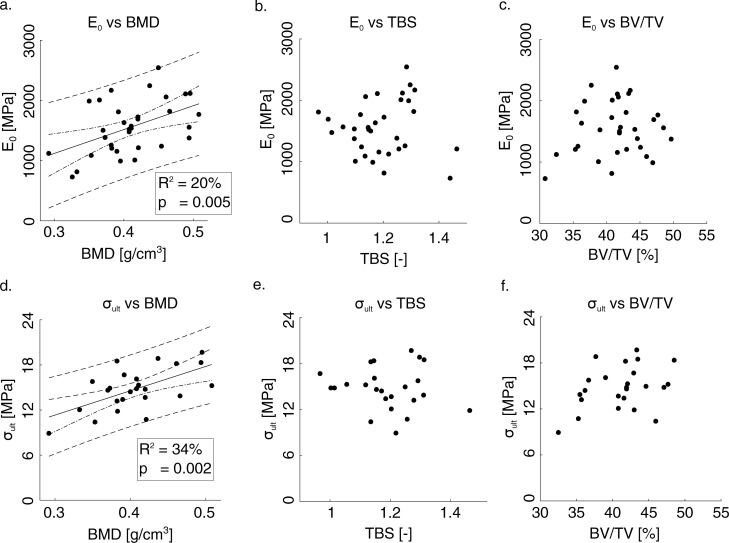
Variation of the elastic stiffness and strength with the BV/TV and other clinical parameters (BMD and TBS). In the case of significant correlation between each parameter, a linear regression line is specified. R^2^ = 0.20; p = 0.005 in a) and R^2^ = 0.34; p = 0.002 in d). The dashed lines are referred to confidence and prediction intervals. Regression lines are added only for significant correlation.

We tried, then, to find a correlation between the clinical, morphometric and mechanical parameters. Fig 3 shows the trend of the mechanical parameters (E_0_ and σ_ult_) with respect to the clinical ones (BMD and TBS) and to a morphometric parameter (BV/TV). It is found a weak correlation of E_0_ with BMD (R^2^ = 0.20; p = 0.005), σ_ult_ and BMD (R^2^ = 0.34; p = 0.002). The correlation with other parameters is provided in Table 1. The yield stress, σ_y_, showed a weak correlation with BMD (R^2^ = 0.23; p = 0.005) and TBS (R^2^ = 0.10, p = 0.046) (Table 1). No significant linear trend was found between the mechanical properties (E_0_ and σ_ult_) and the parameters TBS and BV/TV. The bone strength, measured as ultimate stress (σ_ult_) is linearly related to DA (R^2^ = 0.18, p = 0.02). On the contrary, there is no statistical association between ultimate and yield strain and other morphometric and clinical parameters.

The variation of the elastic modulus (Fig 2A) is the only variable that significantly changes within the different groups (p < 0.001, ANCOVA); in particular, G1% is different from G2%, G3.5%, and G5%, whereas G2%, G3.5%, and G5% show a similar average value. The variation of E is significantly different from zero for G2%, G3.5% and G5% (p < 0.005).

## 4. Discussion

Direct assessment of bone resistance to fracture and damage requires mechanical testing, which is not possible to be included in a clinical protocol. Here, to investigate the effect of damage on bone quality and quantity, we identified four damage levels (as four levels of strain, in both the elastic and the post-yield regions) and we carried out compressive tests on samples of trabecular bone to induce mechanical damage. We analyzed all the parameters that could be involved in the damage process. We used a combination of different characterization techniques to provide a thorough analysis of bone quality and how it is affected by the presence of damage. The effect of damage accumulation in the trabecular bones has been analyzed from different points of view: i) clinical, ii) morphological, and iii) mechanical.

**Fig 4:**
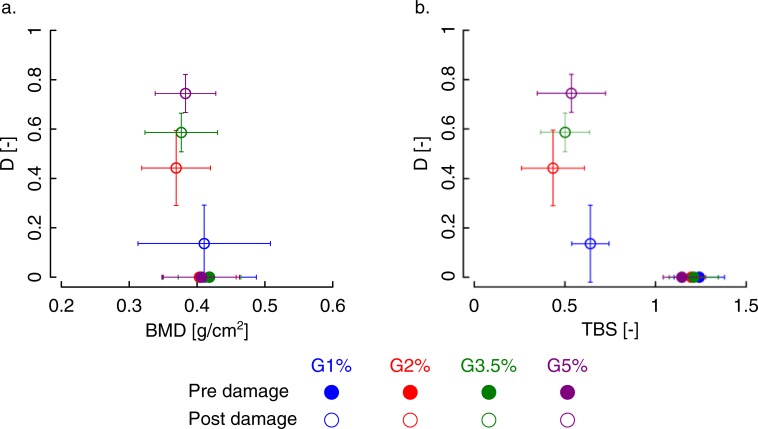
Comparison of accumulated damage obtained from different strain levels with a) BMD and, b)TBS.

From a **clinical point of view**, BMD has shown to be an indicator of bone quantity more than quality, taking into account the presence of mineral and not the presence of damage. Indeed, from statistical analyses, we found significant changes in BMD only for the specimens subjected to damage larger than 3.5% strain (Fig 2B and Fig 4A). However, we should underline that being the resolution of the DXA scanning images not high enough, it was not possible to detect the microcracks propagated in the specimens during the overloading. BMD resulted in the best predictor of mechanical parameters, despite the weak correlation factors.

TBS has shown to be a quality index that is markedly affected by the presence of damage (Fig 2C and Fig 4B). This is consistent with previous evidence of using TBS as a risk factor and its role as a complementary to the BMD [10,14,73]. Our results indeed, exhibit a significant change in the TBS before and after damage, due to the alteration of the microstructure of the trabecular bone under severe mechanical damage loadings. The decrease of TBS is about 50% when the applied strain is larger than 1%. It seems that if the elastic limit is reached, the TBS values are strongly affected by the damage, more than the other morphological parameters, which exhibit significant but smaller variations. However, TBS is not able to distinguish between the different damage levels (Fig 4B). After the first level, corresponding to a loading level close to the yield strain, in fact, the values of TBS are markedly smaller than the values of undamaged specimens but not significantly different from each other. TBS, therefore, seems to be able to identify a damaged bone but not to quantify the damage itself. Furthermore, we did not find any correlation between TBS (before and after damage and their ratios), and damage, meaning TBS (p>0.05) can only qualitatively describe damage but not quantitatively (Fig 4B). There is not any relationship between TBS and plastic strain (p > 0.05) as well.

We also found a significant linear correlation between TBS and the parameters BV/TV. TBS is a 2D representation of a 3D microstructure. In 3D, microstructural changes can be captured by BV/TV, and in 2D, our results show TBS can effectively do the same. Although for the human trabecular bone, it is shown that BV/TV and fabric tensor are a better determinant of stiffness than TBS [74]. DXA was performed on both lumbar spines and trabecular specimens obtained from the spines. The size of the specimens is the largest size that we could obtain within the dimensions of the porcine vertebra. As the aim of this study was to determine the tissue mechanical properties, and how such properties are affected by mechanical damage, we could not perform the test on the whole vertebra. However, we believe that increasing the size of the specimens makes it easier to select the area for the DXA measurements.

Comparison of TBS and BMD for the entire porcine vertebra with the data for human lumbar spine showed that our results are in the same range of the reported data in the literature [9], thus assessing the validity of this study. However, the bone fraction (BV/TV) shows slightly higher value compared to the human vertebra. This difference could come from the rather young porcine samples in our study.

**From a morphological point of view**, we found significant changes in several morphological parameters after yielding, indicating how a higher level of damage affects the microarchitecture.

In particular, we can observe a significant change of BS/TV in damage groups after yielding, i.e. G2% to G5%. It has been shown in [75] that local failure initiates in the sub-regions with smaller BV/TV. This makes smaller total volume, as we also observed in our experiments, which could increase BV/TV and decrease BS/TV. Although we did not find a significant increase for BV/TV, the individual observation of TV for each specimen showed a significant decrease specifically for those specimens that were tested after yielding. We also observed that bone volume for the samples tested after yielding diminishes, balancing the total volume reduction. We also did not find any significant correlation between BV/TV and damage.

Trabecular spacing (Tb.Sp.) also showed a significant reduction for the post-yield groups. This could be justified by microscopic damage and propagation of microcracks in the trabeculae under severe damage loading [53,76–78]. As we performed the μCT scans in the wet condition, the fractured trabeculae could be washed away, producing larger interspacing between trabeculae. We did not perform labeling of microcracks in this study. Therefore we could not measure the size of the microcracks in the post-yield mechanical loading. Owing to the size of microcracks in the trabecular bone, synchrotron CT imaging at the resolution of less than 1μm will be adapt to precisely detect the microcracks in bone [77,79–82].

The degree of anisotropy showed a significant change after damage for groups G3.5% and G5%. This parameter is a scalar parameter that does not provide more information about the orientation of the trabeculae. A more in-depth investigation of the results showed that the largest eigenvalue after and before damage remained the same, whilst, the smallest eigenvalue decreased for the specimens after damage. Based on the definition of MIL [27] and fabric tensor [67], the angle between the largest diameter of the ellipsoid and z-axis did not change after damage, while the angle of two other diameters of the ellipsoid and z-axis changed significantly. This shows that the orientation of the microstructure (trabeculae) changes due to the compressive loading. In other words, the fitted ellipsoid became elongated along its major axis while its minor axis rotated toward the loading direction (z-axis). This can also be inferred from the increase in DA, as it means higher anisotropy in the microstructure of the trabecular bone due to the induced damage.

Trabecular specimens in this study showed a plate-like structure due to visual observation from micro-CT scans. It was reported in the past that damage fraction area is higher in the plate-like rather than rod-like trabecular structures [53,83,84]. Nevertheless, we did not measure such damage fraction area.

**From a mechanical point of view,** the increase of damage level resulted in a stiffness degradation, which is significant above the yield limit. In the last group, where the strain level reaches 5%, we observed about 80% of stiffness degradation due to the microdamage propagation in the trabecular bone. This is in line with the results of compressive damage tests on trabecular bones previously reported in the literature [57,85,86]. The damaged specimens showed barreling and macroscopic shear plane fracture after yielding, similarly to the reported data for human [75] and bovine trabecular bone[61].

The elastic stiffness (R^2^ = 0.2, p = 0.005) and strength (R^2^ = 0.34, p = 0.002) of trabecular porcine in this study were linearly correlated to the bone mineral density (BMD). The strong correlation between these primary mechanical properties and BMD has been reported for the human trabecular bone of proximal femur which were R^2^ = 0.57 for elastic stiffness, and R^2^ = 0.82 for strength [7]. Although we did not find any significant correlation between TBS and primary mechanical properties, i.e., elastic stiffness and strength, a significant linear correlation between TBS and the elastic stiffness for human lumbar vertebrae (R^2^  = 0.41; p = 0.007) has been reported [30]. A significant correlation between bone volume fraction (BV/TV) and elastic stiffness (R^2^ = 0.82, p = 0.002) and strength (R^2^ = 0.87, p < 0.001) were reported for the cubic-shape trabecular bone specimens obtained from porcine vertebrae [37]. Our results do not show such significant correlation between BV/TV and those primary mechanical properties, which can be due to the small variation of BV/TV values of specimens tested in this study. However, the mechanical properties in this study are within the range of ones reported in [37].

One of the limitations of this study is related to the characterization tools that we used for the determination of damage. We quantified damage from the mechanical point of view based on the degradation of stiffness, and we did not characterize it by measuring the damage surfaces/volumes using staining protocols that have been used for micro-CT images [87,88]. Those data could have been used to check the sensitivity of clinical parameters specifically TBS and other morphological parameters in the formation of microcracks.

## 5. Conclusion

Bone’s resistance to fracture depends on several factors: the bone mass, the microarchitecture, including the geometry and distribution of the trabeculae, and the tissue material properties. Here, the adoption of multiple characterization techniques provided a more comprehensive analysis of bone parameters and how they are affected by the presence of damage. The possibility of mechanically induced damage of different entity allowed us to perform a systematic study on the effect of different damage levels on bone tissues, from diverse perspectives (e.g., clinical, morphological, and mechanical).

BMD and TBS have shown to be complementary parameters to assess bone strength, the former assessing the bone quantity, the bone resistance to damage, and its loading sensitivity, and the latter the bone quality and the presence of damage. BMD is slightly affected by the damage (i.e., BMD is only affected by damage level above 3.5% strain), whereas TBS can always be considered as a good indicator of damage, without being able to quantify it though. Moreover, it cannot perfectly explain the risk of fracture in the bone tissue, or how healthy bone is, as we did not find any linear relationship between TBS and the mechanical strength.

The outcome of our DXA analyses is comparable to those provided in the literature for the human lumbar spine [71], proving the similarity of porcine and human trabecular bone from the lumbar spine, and endorsing a more extensive validity of this research.

This study confirms that no single method can provide a complete characterization of bone tissue, and the combination of complementary techniques is required for an accurate and exhaustive description of bone health. Limitations of the current study regard the difficulty of quantifying the damage effect. In future works, we aim to quantify the damage effect, not only from the mechanical perspective but also from the clinical and morphological viewpoints and to monitor the damage evolution. The latter would be possible by performing mechanical testing into a micro-CT scan device. In an ongoing work, we are trying to investigate the effect of damage induced by fatigue loading, coupling experiments with numerical models, able to mimic bone degradation. We believe that numerical models, built from BMD data could provide a valid and versatile tool, easily implemented in the clinical routine, for the assessment of the bone quality and the prediction of the fracture risk.

## Supporting information

S1 Table Supporting information minimal datasheet supplementary document. (XLS)

S2 Table Clinical parameters before and after the mechanical testing. (PDF)

S3 Table Morphometric properties of the trabecular porcine specimen before damage testing. (PDF)

S4 Table Mechanical properties of trabecular porcine specimens. (PDF)

## Abbreviations

A_0_ [mm^2^]: Primary Area
BMD: Bone Mineral Density
BS [mm^2^]: Bone Surface
BS/TV [1/mm]: Bone Surface to Total Volume
BV/TV [–]: Bone Volume to Total Volume
D [–]: Damage
DA [–]: Degree of Anisotropy
DXA: Dual X-Ray Photon Absorptiometry
E_0_ [MPa]: Initial Elastic Stiffness
E [MPa]: Unloading Elastic Stiffness
F [N]: Force
M [–]: Fabric Tensor
m_i_ [–]: Eigenvalues
*m*_*i*_ [–]: Eigenvectors
MIL [–]: Mean Intercept Length
S [mm]: Stroke
TBS: Trabecular Bone Score
Tb. N [–]: Trabecular Number
Tb.Sp.[mm]: Trabecular Spacing
Tb.Th [mm]: Trabecular Thickness
ε [%]: Strain
ε_y_ [%]: Yield Strain
ε_ult_ [%]: Ultimate Strain
μCT: Micro- computed Tomography
ρ_p_: Porosity
ρ_s_ [–]: Bone Relative Density
σ [MPa]: Normal Stress
σ_y_ [MPa]: Yield stress
σ_ult_ [MPa]: Ultimate stress
