# Supplementary material for: Determinants of bone damage: An ex-vivo study on porcine vertebrae
Source: PLoS One. 2018 Aug 16;13(8):e0202210. doi: 10.1371/journal.pone.0202210 (PMC6095531; doi:10.1371/journal.pone.0202210)
Supplement: S2 Table — (PDF) [file pone.0202210.s002.pdf]

S1 Table. Clinical parameters before and after the mechanical testing. Results are presented as a mean ± standard deviation. A significant change has been observed for all parameters before and after mechanical loading.

Clinical properties	Before damage	After damage
Bone Mineral Density, BMD [g/cm^3]	0.41 ± 0.05	0.38 ± 0.06
Trabecular Bone Score [-]	1.19 ± 0.11	0.52 ± 0.17
