# Supplementary material for: Determinants of bone damage: An ex-vivo study on porcine vertebrae
Source: PLoS One. 2018 Aug 16;13(8):e0202210. doi: 10.1371/journal.pone.0202210 (PMC6095531; doi:10.1371/journal.pone.0202210)
Supplement: S3 Table — (PDF) [file pone.0202210.s003.pdf]

S2 Table. Morphometric properties of the trabecular porcine specimen before damage testing. Data are presented as a mean ± standard deviation. Mean, and the standard deviation was calculated from the pooled data as no significant difference was found between morphological parameters of samples between different vertebra locations.

Morphometric properties	Value
Bone volume to total volume (BV/TV) [%]	41.38 ± 4.54
Trabecular Thickness (Tb. Th.) [mm]	0.26 ± 0.12
Trabecular Spacing (Tb. Sp.) [mm]	0.44 ± 0.17
Bone surface to bone volume (BS/BV) [1/ mm]	10.64 ± 0.99
Bone surface to total volume (BS/TV) [1/mm]	4.37 ± 0.35
Degree of Anisotropy (DA)	0.56 ± 0.09
m_1	0.60 ± 0.10
m_2	1.03 ± 0.11
m_3	1.38 ± 0.07
