# Supplementary material for: Determinants of bone damage: An ex-vivo study on porcine vertebrae
Source: PLoS One. 2018 Aug 16;13(8):e0202210. doi: 10.1371/journal.pone.0202210 (PMC6095531; doi:10.1371/journal.pone.0202210)
Supplement: S4 Table — (PDF) [file pone.0202210.s004.pdf]

S3 Table. Mechanical properties of trabecular porcine specimens. No significant difference was found between mechanical properties within vertebra for different spines. Therefore, mechanical properties were pooled and presented as a mean ± standard deviation.

Mechanical properties	Value
Initial elastic modulus, E_0 [MPa]	1558.90 ± 441.59
Yield stress, σ_y [MPa]	12.43 ± 3.37
Yield strain, ε_y [%]	1.03 ± 0.17
Strength, σ_{ult} [MPa]	14.76 ± 2.78
Ultimate strain, ε_{ult} [%]	1.71 ± 0.49
